# Platelet-rich plasma enhances post-conditioning recovery from testicular ischemia–reperfusion injury: a novel experimental approach

**DOI:** 10.1038/s41598-026-46712-6

**Published:** 2026-04-17

**Authors:** Amena-Elmongy, Gannah-Samy, Alaa Samy, Zeinab Shouman, Aya Megahed, Gamal Karrouf

**Affiliations:** 1 Department of Surgery, Anesthesiology, and Radiology, Faculty of Veterinary Medicine, Mansoura University, Mansoura, 35516 Egypt; 2 Department of Cytology and Histology, Faculty of Veterinary Medicine, Mansoura University, Mansoura, 35516 Egypt; 3 Faculty of Health Science Technology, Mansoura National University, Gamasa, Egypt; 4 Department of Clinical Pathology, Faculty of Veterinary Medicine, Mansoura University, Mansoura, 35516 Egypt

**Keywords:** Ischemia reperfusion injury, Post-conditioning, Platelet-rich plasma, Oxidative stress, Apoptosis, Cell biology, Diseases, Medical research, Urology

## Abstract

The present study aimed to investigate the protective effects of platelet-rich plasma combined with post-conditioning on testicular ischemia–reperfusion injury in a rat model. Thirty-two adult male rats were randomly assigned to four groups (n = 8 each): Sham (SH), Ischemia–reperfusion (I/R), post-conditioning (PC), and platelet-rich plasma-post conditioning PRP/PC. Testicular torsion was induced for 3 h, followed by 24 h of reperfusion. After 24 h, blood and testicular tissues were collected for histopathological and immunohistochemical analyses. Ischemia–reperfusion notably increased oxidative stress, apoptosis, and inflammatory markers, as shown by higher levels of malondialdehyde, caspase-3, TNF-α, and IL-6 (*p* < 0.05). These changes were reduced by PC and more effectively by PRP/PC. Antioxidant markers, including catalase, reduced glutathione (*p* < 0.0001), eNOS, HSP70, and VEGF (*p* < 0.0001), were significantly higher in the treated groups than in the I/R group. Histological analysis demonstrated improved testicular structure, with Johnsen’s scores of 8–10 (SH), 2–3 (I/R), 6–9 (PC), and 4–9 (PRP/PC). Notably, PRP/PC showed increased expression of Bax and NF-κB. Hematological results revealed significant changes in Hb, RBCs, and HCT, while MCHC and MCH remained unchanged, and MCV showed minor differences. The data of the present study indicated that combined with PC provided partial protection against testicular I/R injury, reducing oxidative stress, apoptosis, and inflammation while preserving testicular architecture. These findings suggest early restorative effects rather than complete functional recovery.

**Supplementary Information:**

The online version contains supplementary material available at 10.1038/s41598-026-46712-6.

## Introduction

Testicular torsion is a urological emergency in which twisting of the spermatic cord leads to ischemia of the testis, and subsequent surgical detorsion can cause ischemia–reperfusion (I/R) injury, which exacerbates tissue damage through oxidative stress, inflammation, and apoptosis^1^. The pathophysiology of I/R injury involves the generation of reactive oxygen species (ROS), lipid peroxidation, neutrophil infiltration, and the activation of pro-inflammatory cytokines, all of which contribute to germ cell loss and can impair both the affected and contralateral testis^2^. I/R injury after testicular torsion is significant because it increases the risk of long-term complications such as reduced sperm count, decreased sperm motility, and potential infertility, even if the testis is surgically salvaged^3^.

Current therapeutic strategies for I/R injury primarily focus on prompt surgical detorsion to restore blood flow. Still, this intervention alone does not prevent further tissue damage caused by oxidative stress, inflammation, and apoptosis during reperfusion^1^. Numerous experimental therapies have been investigated, including antioxidants, anti-inflammatory agents, calcium channel blockers, hormones, and novel drugs such as varenicline and avanafil, all of which have shown protective effects in animal models by reducing oxidative stress, inflammation, and cell death^4–6^. Several natural compounds have been investigated for their protective role against testicular ischemia–reperfusion injury. Previous experimental studies have investigated various pharmacological and natural agents to attenuate testicular ischemia–reperfusion injury. Compounds such as Visnagin, Bromelain, Arbutin, Proanthocyanidin, and Avanafil have demonstrated protective effects through antioxidant, anti-inflammatory, anti-apoptotic, and inflammasome-modulating mechanisms, including regulation of the NLRP3 pathway^7–11^.

Despite promising results in preclinical studies, the main limitation is that most of these therapies have not been translated into routine clinical practice due to a lack of large-scale human trials, uncertain optimal dosing, and potential side effects^12^. Additionally, the complexity of I/R injury mechanisms means that single-agent therapies may be insufficient, and combination treatments or new approaches like ischemic postconditioning are being explored but remain experimental^12^. Ischemic postconditioning (IPostC) and remote ischemic postconditioning (RIPostC) are emerging strategies to reduce testicular damage following I/R injury, which have been shown to decrease oxidative stress, inflammation, and apoptosis, and to preserve testicular structure and function^13,14^.

Platelet-rich plasma (PRP) has shown protective and therapeutic effects against testicular I/R injury in animal models, as PRP is rich in growth factors such as PDGF, TGF-β, VEGF, and IGF-1, which promote angiogenesis, modulate inflammation, and support extracellular matrix remodeling^15^. Anti-inflammatory cytokines in PRP also reduce oxidative stress, inflammation, and apoptosis, improve antioxidant enzyme activity, and help preserve testicular structure and function after torsion–detorsion^16^. Studies report that PRP administration can restore testosterone levels, improve spermatogenesis, and enhance sperm quality after I/R injury^17^. Both PRP and PC act through complementary mechanisms to enhance tissue repair and regenerative signaling by progenitor activation and paracrine factor release^18^^,^^19^. This study hypothesizes that the combined administration of PRP and PC will produce superior regenerative and protective effects compared with either treatment alone. To our knowledge, this study is the first to combine surgical intervention via PC with intratesticular administration of PRP.

## Materials and methods

### Animals and study design

Thirty-two healthy adult male albino rats, with a mean body weight of 250–300 g and aged 10–12 weeks, were used. The animals were housed under standard laboratory conditions at 22 ± 2 °C, with a relative humidity of 65–70% and a 12-h light/12-h dark cycle. All experimental procedures were conducted at the Medical Experimental Research Center, Faculty of Medicine, Mansoura University, Mansoura, Egypt. Animals were acclimated to the housing conditions for 2 weeks before the experiment. They received a standard laboratory diet from the Department of Nutrition, Faculty of Veterinary Medicine, Mansoura University, Egypt, with free access to food and water.

The experimental protocol was approved by the Research Ethics Committee of the Faculty of Veterinary Medicine at Mansoura University, Egypt (registration code: MU‐ACUC (VM.MS.23.10.90). All animal procedures were conducted in accordance with the relevant guidelines and regulations.

**Fig. 1:**
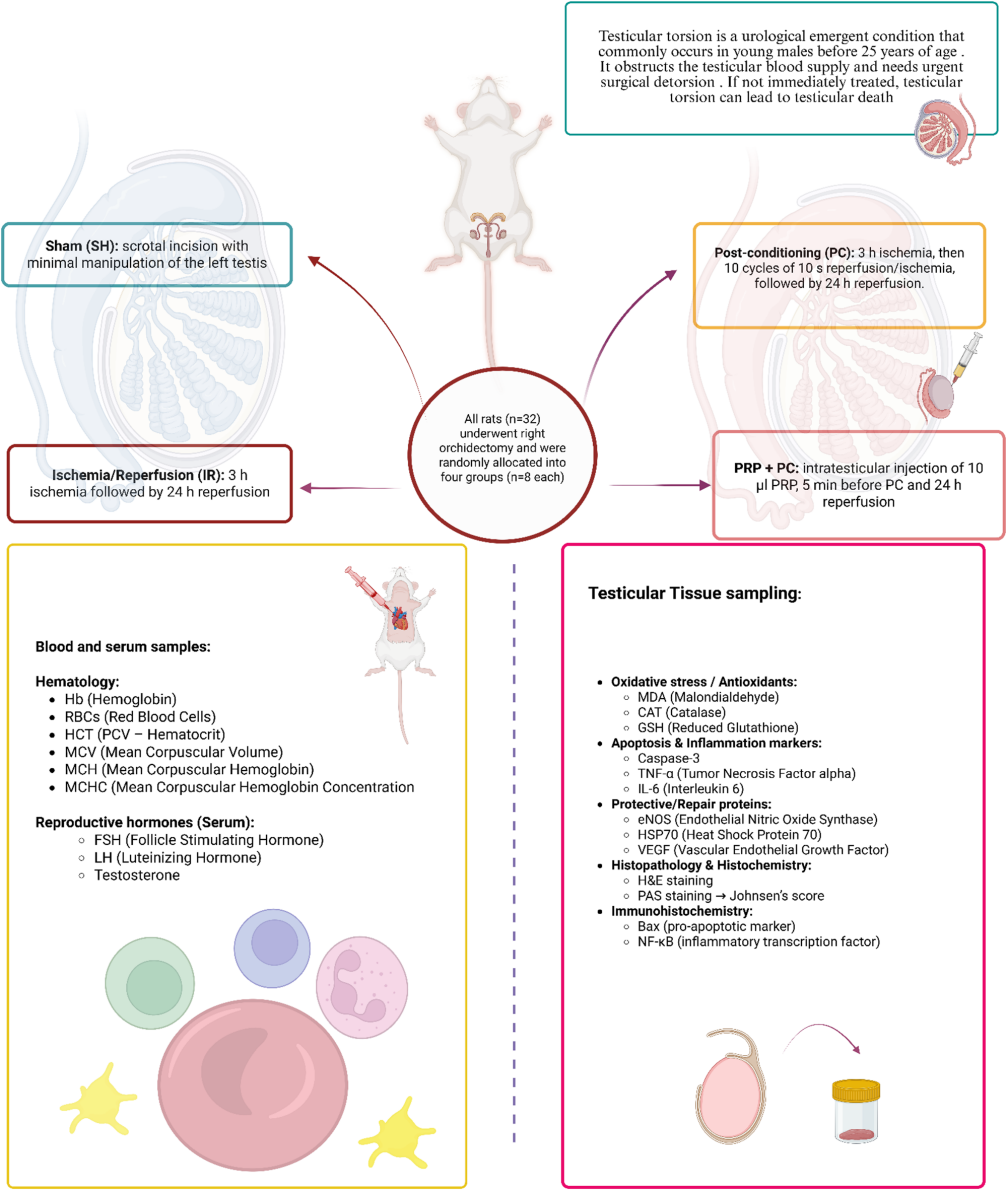
Schematic representation of the experimental design and the methods of evaluation, created by the author using free tools in Biorender.com.

A right orchidectomy was performed in all rats, and then they were randomly allocated into four groups, n = 8 per group, based on the intervention applied to the left tests:

- SH (Sham): only scrotal incision and gentle manipulation of the left tests without further intervention.
- I/R: 3 h of ischemia followed by 24 h of reperfusion.
- PC: 3 h of ischemia followed by 10 cycles of reperfusion (10 s) and ischemia (10 s), then 24 h of reperfusion.
- PRP/PC: intratesticular injection of PRP (10 µl/rat) administered 5 min before PC, followed by 24 h of reperfusion (Fig. 1).

### Platelet-rich plasma preparation

The PRP was prepared, and platelet counts were determined according to the method of^20^ and^21^. Briefly, blood was drawn from the right ventricle of pre-anesthetized eight donor rats into tubes containing 4% sodium citrate. Samples were centrifuged at 1,480 rpm for 6 min to separate PRP and platelet-poor plasma (PPP) from other cellular components. The greyish PRP layer was collected, transferred to a clean tube, and centrifuged again at 3,400 rpm for 15 min to concentrate platelets further. Two-thirds of the supernatant containing PPP was discarded, and the remaining PRP was retained. The final platelet count was 1,950,000 platelets/μl.

### Surgical procedure

Under a complete aseptic preparations and general anesthesia protocol experimental surgery were induced by 10 mg/kg xylazine (ROMBOGESC, EVA CO, EGYPT) and 80 mg/kg ketamine (KETAMAX, INDIA), both were given IP, right unilateral orchidectomy was conducted to all groups while in left testes of all except sham rats- were applied to testicular torsion through scrotal incision and exteriorization of the testes then rotating the spermatic cord 720^ο^ clockwise subsequently was fixed with scrotal skin using 4/0 proline 3 h for ischemia afterward repositioning to normal anatomical position 24 h later as a reperfusion, while post-conditioning technique was applied only in PC and PRP/PC immediately at reperfusion in a manner of 10 episodes of 10 s reperfusion and 10 s ischemia while PRP was administered 10 µl/rat i.t five minutes before post-conditioning according to Samy et al.^22^.

### Euthanasia and sampling

Euthanasia After 24 h of reperfusion, which was performed by 120 mg/kg, i.p of Thiopental Na (THIOPENTAL INJECTION BP 2019, EPICO, EGYPT)^23^.

Blood was collected via Cardiac Puncture. Two separate blood samples were collected. The first sample was collected in Eppendorf tubes containing K2EDTA (0.5 mg/ml blood) and was well mixed to obtain a complete blood picture. The second sample was collected without an anticoagulant, then centrifuged at 1500 rpm for 10 min, and the serum was collected and cooled at -20 °C for reproductive analysis.

Testicular tissue was divided into three pieces and manipulated for different measurement purposes; The first part (0.5 g) were crushed in 5 ml ice-cold PBS (PH 7.5) and centrifuged at 2000 rpm for 15 min at 4 °C, supernatant was carefully aspirated in and were frozen at -20 °C for estimation of oxidative (MDA), antioxidative (CAT and GSH), apoptotic (Caspase3), inflammatory (IL 6, TNF α) biomarkers as well as proteins detection (eNOS, VEGF and HSP70), the last part was fixed using 10% neutral buffered formalin for histopathological and immunohistochemical assay.

### Laboratory analyses

#### Biochemical and hematological analyses

Reproductive hormones, including follicle-stimulating hormone (FSH), luteinizing hormone (LH; DCM009-11), and testosterone (DCM002-9), were measured using sandwich ELISA kits (DIAMETRA, Italy) according to the manufacturer’s instructions.

Oxidative stress and antioxidant markers, including malondialdehyde (MDA; cat. No. Md 25 29), catalase (CAT; cat. No. Ca 25 17), and reduced glutathione (GSH; cat. No. Gr 25 11), were evaluated using enzymatic colorimetric assays.

Hematological parameters, including hemoglobin (HB), hematocrit (HCT/PCV), mean corpuscular volume (MCV), mean corpuscular hemoglobin (MCH), and mean corpuscular hemoglobin concentration (MCHC), were measured using a Mindray BC-20 Vet analyzer.

Apoptotic and inflammatory markers, including NOS3/eNOS, heat shock protein 70 (HSP-70), vascular endothelial growth factor (VEGF), Caspase-3, and tumor necrosis factor-alpha (TNF-α), were determined using commercially available ELISA kits (cat. Nos. RTFI00087, CSB-E08308r, EK0540, CSB-E08857r, and R6000B).

#### Histopathological and immunohistochemical examination

Testis samples were collected, fixed in 10% neutral buffered formalin, processed, embedded in paraffin, and sectioned at 3 µm thickness. Sections were stained with hematoxylin and eosin (H&E) according to Mondal^24^, Fischer et al.^25^, and with periodic acid–Schiff (PAS) according to Wahyuni et al.^26^.

Immunohistochemical analysis of NF-κB and Bax proteins in paraffin-embedded testis sections was performed according to the method described by Renshaw^27^. Briefly, sections were deparaffinized, rehydrated, and endogenous peroxidase activity was blocked. Sections were incubated with 5% BSA to prevent nonspecific binding, followed by overnight incubation at 4 °C with monoclonal rabbit anti-NF-κB or anti-Bax antibodies. After incubation with HRP-conjugated secondary antibody, immunoreactivity was visualized using DAB, and sections were counterstained with hematoxylin, dehydrated, and mounted. The expression of NF-κB and Bax proteins was analyzed in 100 randomly selected seminiferous tubules per rat, as described by Delfino and Walker^28^ The expression of Bax and NF-κB was examined using threshold analysis in which the ratio of DAB positivity in the seminiferous tubule was compared to the tissue section area, and the obtained ratios were converted to a percentage^29^.

#### Morphometric analysis

Certain parameters were measured after examination of the stained sections, including seminiferous tubule diameter (SD), seminiferous epithelium thickness (ET), and luminal area width (LW)^30^. The images were captured and analyzed by ImageJ software. 10–50 tubules from each one of the examined groups were assessed and scored from 1 to 10 according to the severity of tubular injury^31^.

### Statistical analysis

An ordinary one-way analysis of variance (ANOVA) was used to examine the data, with results presented as mean ± standard deviation. Data was tested for normality using the Shapiro–Wilk test, *p* > 0.05, and homogeneity of variance using Bartlett’s test before the ANOVA test. The values were entered into GraphPad Prism version 8.4.3 (GraphPad Software, San Diego, CA, USA; https://www.graphpad.com/scientific-software/prism/) and SPSS (version 22; USA), and Tukey post hoc multiple comparisons were performed thereafter. The results were deemed statistically significant at *p* < 0.05**.**

The sample size was selected in accordance with previous studies using comparable models and endpoints, which reported adequate statistical power to detect treatment-related differences while minimizing animal use in compliance with the 3Rs principle^32^.

## Results

### Histological results

**Fig. 2:**
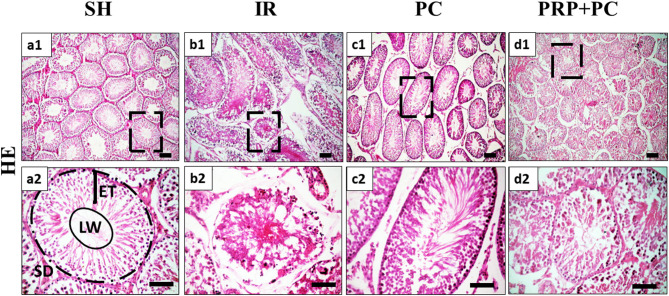
Photomicrograph of HE-stained testicular sections of Sham group (**a1**, **a2**), I/R group (**b1**, **b2**), PC group (**c1**, **c2**), and PRP + PC group (**d1**, **d2**). ET = seminiferous epithelium thickness, SD = seminiferous tubule diameter, LW = luminal width, Scale bars 100 µm (**a1**-**d1**) and 50 µm (**a2**-**d2**).

Hematoxylin and Eosin-stained sections from the sham group showed preserved tissue architecture with normal complete spermatogenesis (Fig. 2a1, a2). Furthermore, the I/R group exhibited depleted seminiferous tubules with vacuolated epithelium, resulting in a significantly increased epithelial thickness (Fig. 2b1, b2 and 6a).

The PC group exhibited seminiferous tubules with morphology similar to the sham group (Fig. 2c1, c2). Meanwhile, the PRP/PC group exhibited variable histological features of the seminiferous tubules; some tubules demonstrated nearly complete spermatogenesis, while others contained a narrow lumen with necrotic cells. Overall, the tubules in this group had a significantly smaller diameter compared to those in the other groups. (Figs. 2d1, d2 and 6b, c).

### PAS staining and Johnsen’s scoring

**Fig. 3:**
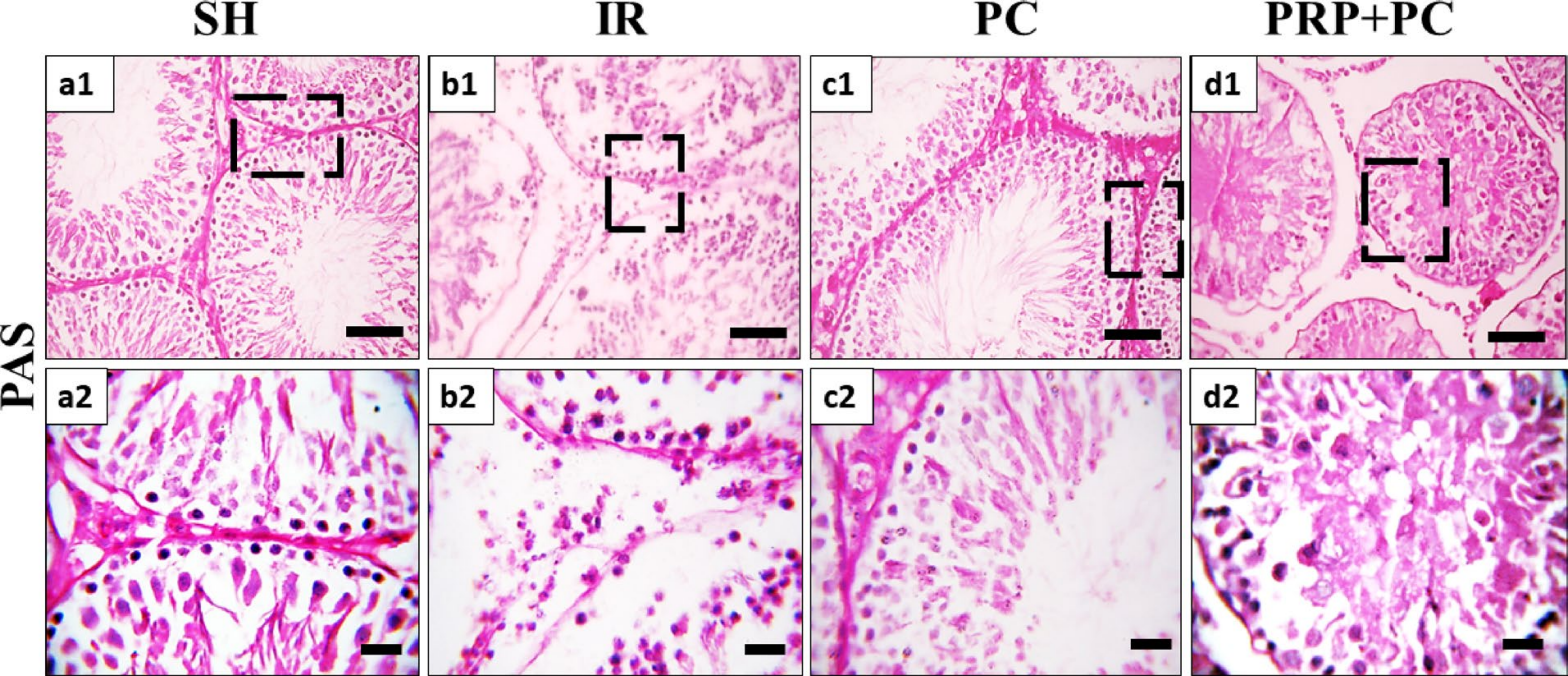
Photomicrograph of PAS-stained testis of Sham group (**a1**, **a2**), IR group (**b1**, **b2**), PC group (**c1**, **c2**), and PRP + PC group (**d1**, **d2**). Scale bars 50 µm (**a1**-**d1**) and 20 µm (**a2**-**d2**).

Using PAS staining to identify different sperm-producing cells, the sham group typically scored high (8–10), indicating complete and abundant sperm production with the presence of numerous or few spermatozoa (Figs. 3a1, a2 and 4). The I/R group mostly scored low (2–3), showing only early sperm cells or no germ cells at all, with occasional tubules reaching the spermatid stage (6–7) (Figs. 3b1, b2 and 4).

**Fig. 4:**
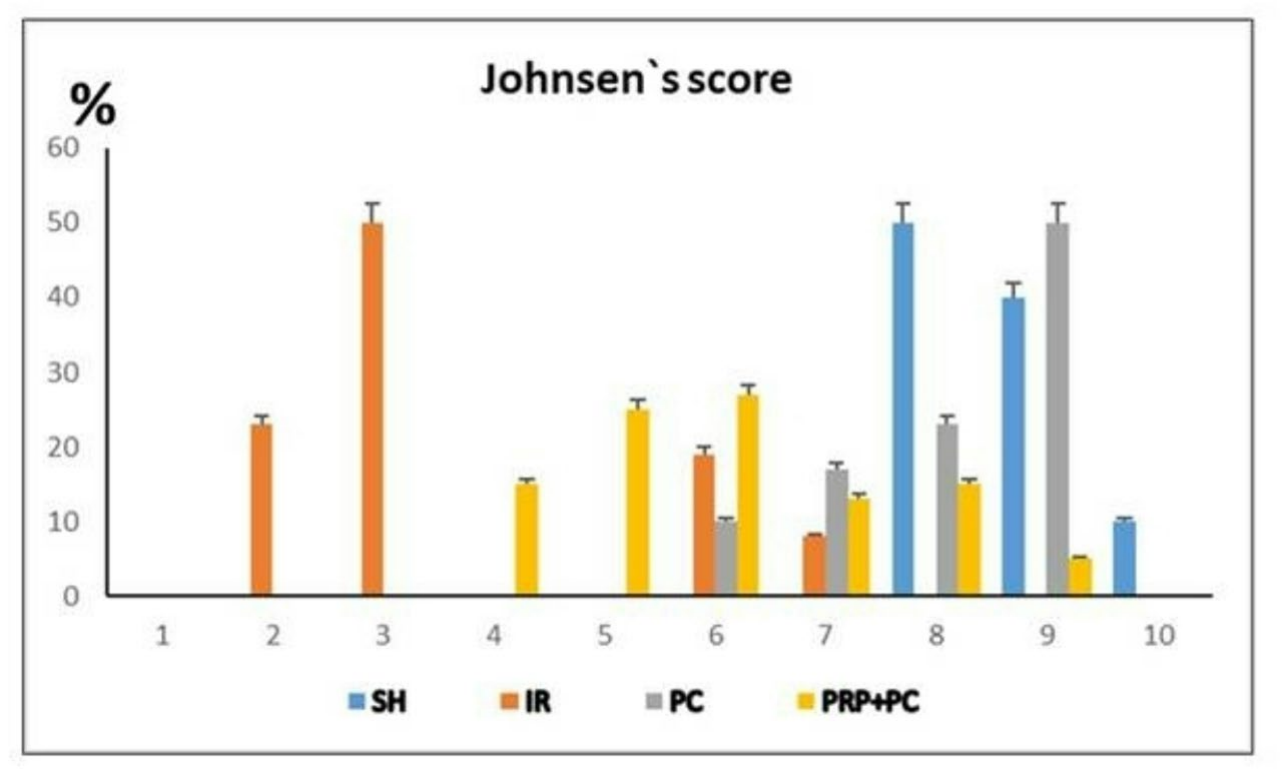
Graph representing the percentage (%) of Johnsen’s score of seminiferous tubules of the tested groups; the result was not significant.

The PC group had scores ranging from 6 to 9, meaning some tubules had almost complete sperm production while others only reached the spermatid level (Figs. 3c1, c2 and 4). The PRP + PC group demonstrated scores ranging from 4 to 9, with most tubules showing spermatogenic progression up to the spermatocyte level, and a few containing spermatids. Some tubules progressed further, showing development up to the spermatozoa stage (Figs. 3d1, d2 and 4).

### Immunohistochemical analysis results

**Fig. 5:**
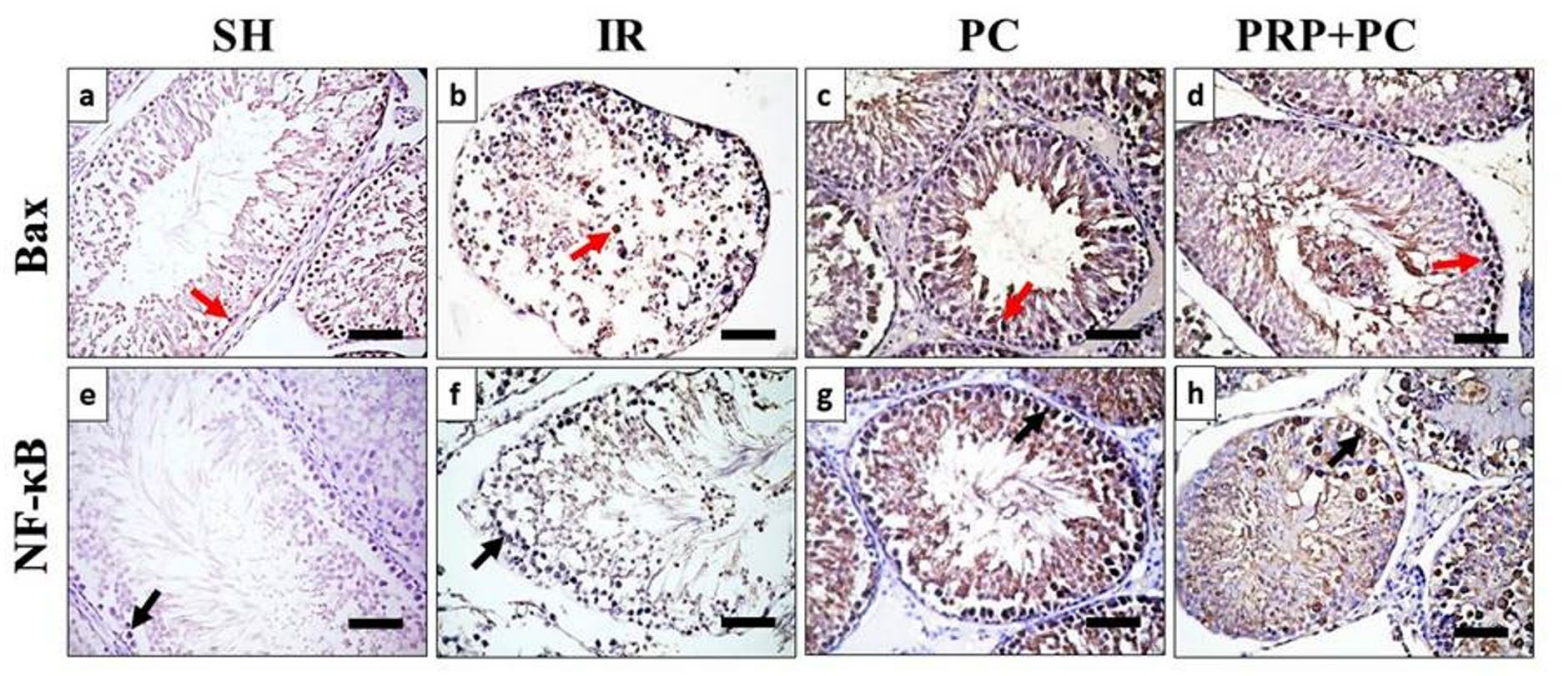
Photomicrograph of immunohistochemically stained testis sections against Bax marker (**a**–**d**) and NF-κB marker (**e**–**h**) in the tested groups. Red arrows = Bax + cells, black arrows NF-κB + cells. Scale bars 50 µm.

**Fig. 6:**
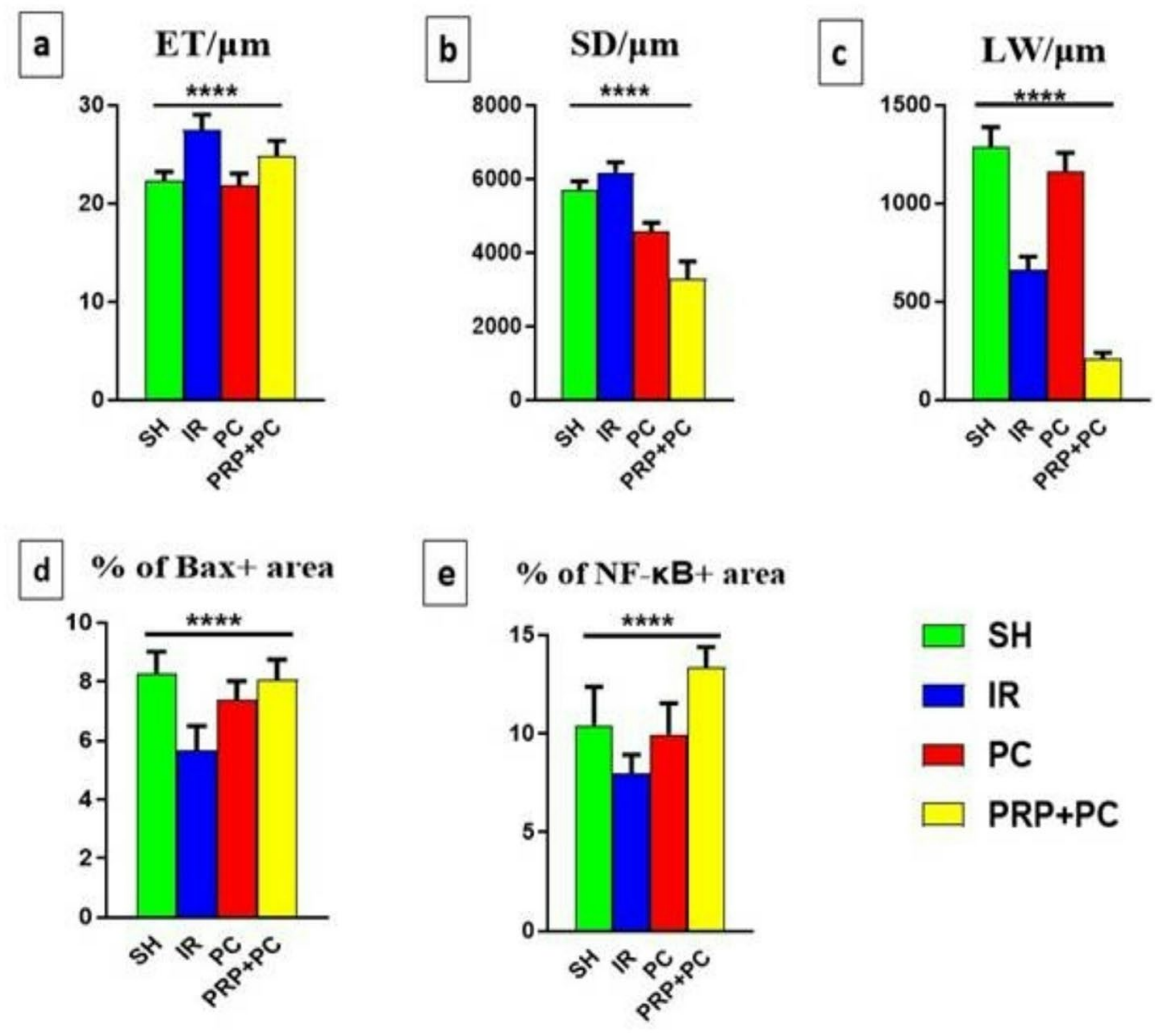
Graphs presenting variable measurements of seminiferous tubules of tested groups, including seminiferous epithelium thickness/µm (**a**), seminiferous tubule diameter/µm (**b**), luminal width/µm (**c**), % of Bax + area (**d**), % of NF-κB + area (**e**). P-value less than 0.01 (highly significant = ****).

Immunohistochemically stained sections for Bax (Fig. 5a–d) and NF-κB (Fig. 5e–h) were analyzed to assess their expression levels, indicated by brown staining in the nuclei or cytoplasm of spermatogenic cells. The PRP + PC group exhibited high expression levels of both markers, indicating increased apoptotic activity (Bax) and heightened inflammatory or stress response NF-κB within the testicular tissue (Fig. 6 d, e).

### Apoptosis and inflammation analysis

**Fig. 7:**
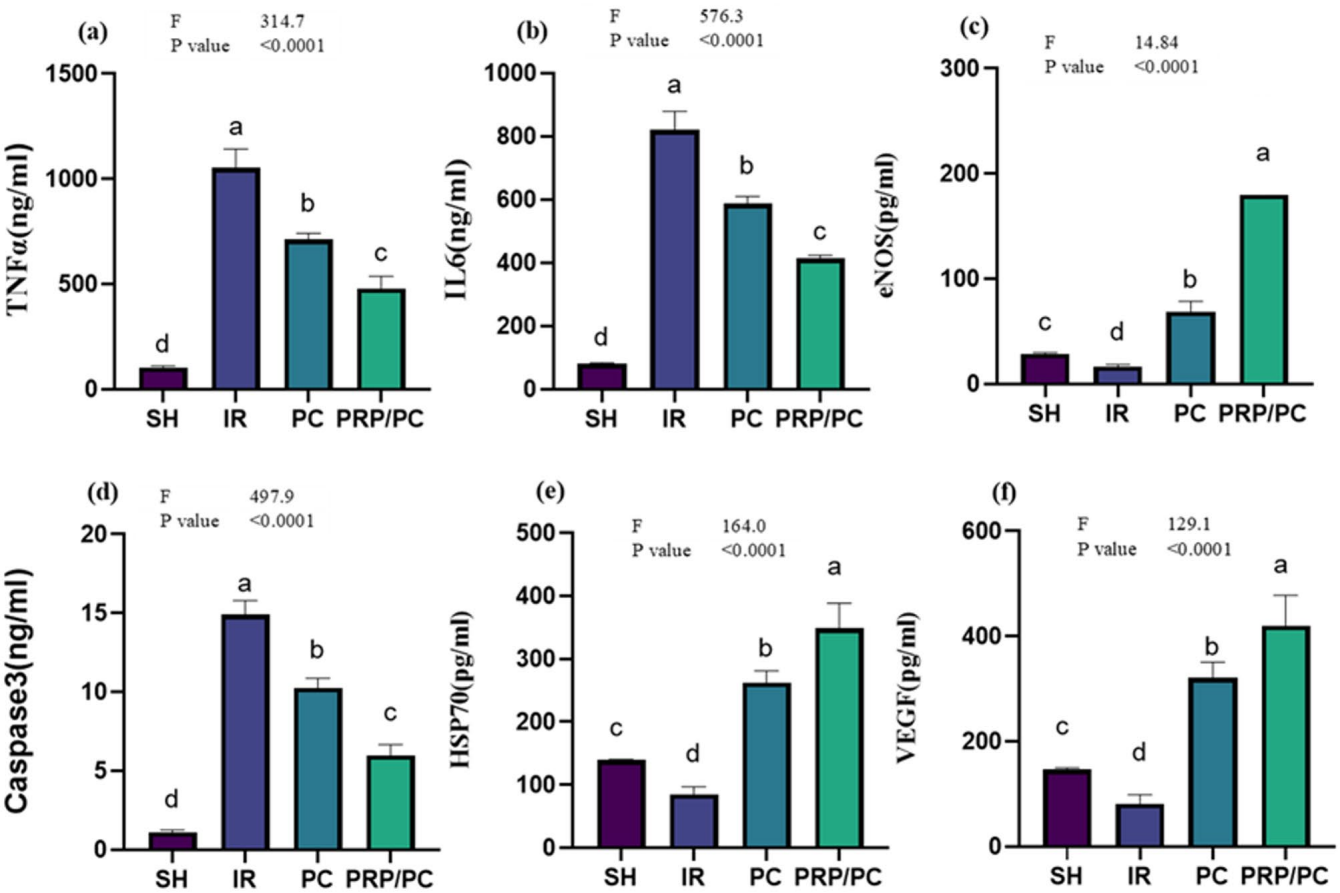
Apoptosis and inflammation analysis. Levels of (**a**) TNFα, (**b**) IL-6, (**c**) eNOS, (**d**) Caspase-3, (**e**) HSP70, and (**f**) VEGF were measured in testis homogenates from the various experimental groups. Data are presented as mean ± SD for each group. Statistical analysis was performed using ordinary one-way ANOVA followed by Tukey’s post hoc test in GraphPad Prism. Different letters (a, b, c, and d) denote statistically significant differences between groups (*p* < 0.05).

The IR group showed a significant elevation in TNFα and IL6 (p < 0.0001) compared with PRP/PC and PC (*p* < 0.0001), respectively, while there was little significant difference between treatment groups, and SH proved the efficacy of PRP and PC on inflammation control. On the other hand, eNOS was reduced in I/R than PRP/PC and PC in comparison with the SH group (*p* < 0.0001), while in focusing on Caspase3 I/R group showed a significant elevation (*p* < 0.0001) compared with PRP/PC and PC (*p* < 0.0001), respectively, while there was little significant difference between treatment groups and SH. PC and PRP/PC showed increased HSP70 levels compared to I/R when compared with the SH rats (*p* < 0.0001), and the level of VEGF was reduced in IR than SH, while improved in PC and PRP/PC (*P* < 0.0001) Fig. 7 and Table 1.

**Table 1:** Apoptosis and inflammation parameters. Concentrations of TNFα, IL-6, eNOS, Caspase-3, HSP70, and VEGF in testis homogenates from different study groups are presented as mean ± SD.

Variables	Groups				F Test	P value
	SH	IR	PC	PRP/PC		
TNF α	104.4 ± 7.01^d^	1054 ± 88.18^a^	713.3 ± 28.4^b^	477.9 ± 59.4^c^	314.7	< 0.0001
IL6	80.72 ± 2.95^d^	820.6 ± 58.4^a^	587.2 ± 22.58^b^	413 ± 1.76^c^	576.3	< 0.0001
eNOS	28.61 ± 1.22^c^	16.54 ± 2.08^d^	68.95 ± 9.63^b^	180 ± 94.14^a^	14.84	< 0.0001
Caspase3	1.131 ± 0.12^d^	14.9 ± 0.89^a^	10.26 ± 0.62^b^	5.983 ± 0.67^c^	497.9	< 0.0001
HSP70	139.5 ± 1.03^c^	85.02 ± 11.89^d^	262.4 ± 18.49^b^	348.7 ± 39.9^a^	164	< 0.0001
VEFG	146.7 ± 2.92^c^	81.78 ± 16.88^d^	321.3 ± 29.58^b^	420 ± 57.76^a^	129.1	< 0.0001

Statistical analysis was conducted using ordinary one-way ANOVA followed by Tukey’s post hoc test with GraphPad software. Different superscript letters (a, b, c, and d) denote statistically significant differences between groups (*p* < 0.05).

Mentioned superscript letters on different groups (raw) are significant at *P* < 0.05 using one-way ANOVA test and Tukey Post hoc test at DFN and DFD equal 3 and 20.

### Oxidative stress and antioxidation evaluation

**Fig. 8:**
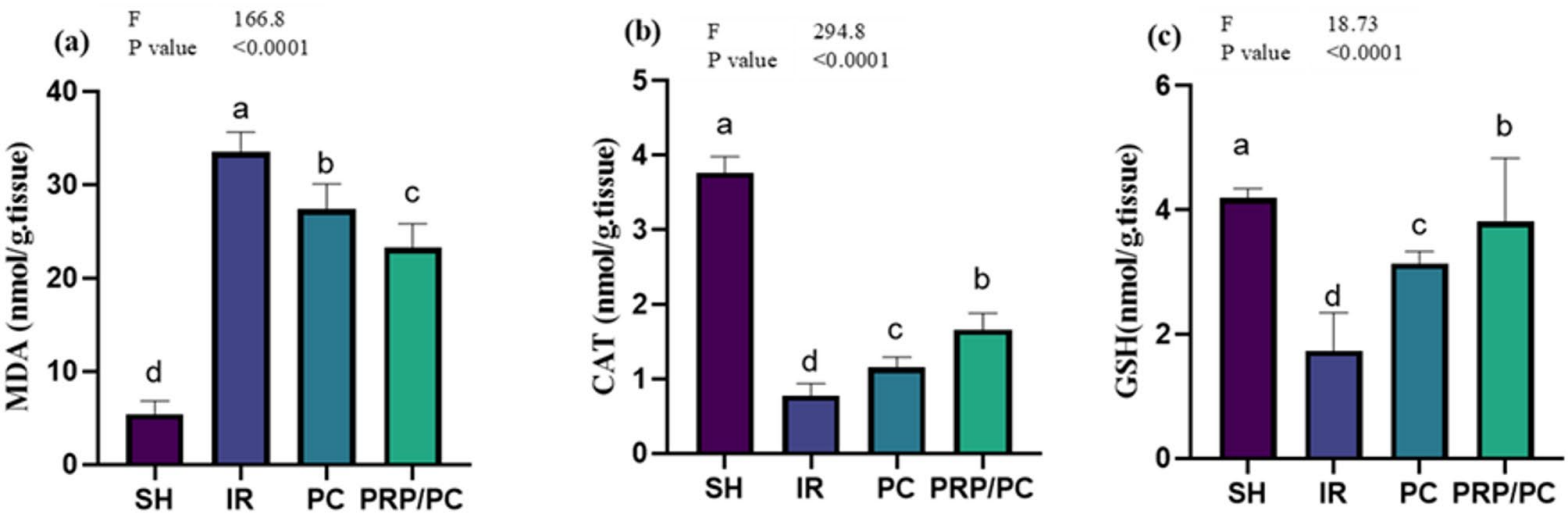
Oxidative stress and antioxidant parameters. Concentrations of (**a**) MDA, (**b**) CAT, and (**c**) GSH in testis homogenates from different study groups are presented as mean ± SD. Statistical analysis was performed using ordinary one-way ANOVA followed by Tukey’s post hoc test with GraphPad software. Different superscript letters (a, b, c, and d) denote statistically significant differences between groups (*p* < 0.05).

The significance of MDA was higher in I/R than in PC and PRP/PC compared with SH rats (*p* < 0.0001), in contrast with CAT and GSH, which were lower in IR than PC, PRP/PC, and SH, respectively (*p* < 0.0001). Figure 8 and Table 2.

**Table 2:** Oxidative stress and antioxidant parameters. Concentrations of (a) MDA, (b) CAT, and (c) GSH in testis homogenates from different study groups are presented as mean ± SD.

Variables	Groups				F Test	P value
	**SH**	**IR**	**PC**	**PRP/PC**		
MDA	5.422 ± 1.4^**d**^	33.49 ± 2.1^**a**^	27.37 ± 2.7^**b**^	23.2 ± 2.6^**c**^	166.8	< 0.0001
CAT	3.767 ± 0.2^**a**^	0.7767 ± 0.16^**d**^	1.15 ± 0.14^**c**^	1.66 ± 0.22^**b**^	294.8	< 0.0001
GSH	4.187 ± 0.15^**a**^	1.737 ± 0.6^**d**^	3.145 ± 0.18^**c**^	3.809 ± 1^**b**^	18.73	< 0.0001

Statistical analysis was performed using ordinary one-way ANOVA followed by Tukey’s post hoc test with GraphPad software. Different superscript letters (a, b, c, and d) denote statistically significant differences between groups (*p* < 0.05).

Mentioned superscript letters on different groups (raw) are significant at *P* < 0.05 using one-way ANOVA test and Tukey Post hoc test at DFn and DFd equal 3 and 20.

### Reproductive hormonal assay

**Fig. 9:**
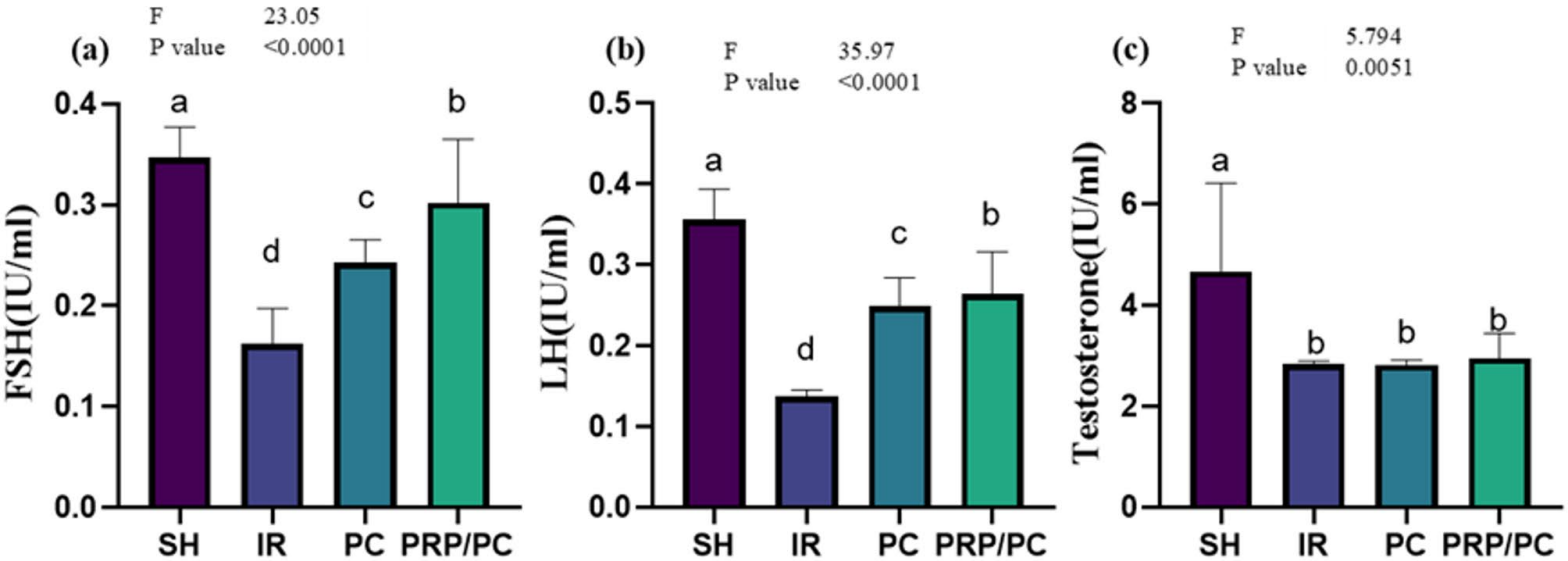
Reproductive parameters. Serum concentrations of (**a**) FSH, (**b**) LH, and (**c**) Testosterone in different study groups are presented as mean ± SD. Statistical analysis was performed using ordinary one-way ANOVA followed by a post hoc test with GraphPad software. Different superscript letters (a, b, c, d) indicate statistically significant differences between groups (*p* < 0.05).

FSH and LH hormones showed significantly lower levels in IR than PC and PRP/PC in comparison to SH (*p* < 0.0001). On the other hand, there was no significant difference between the three groups’ opposition to the SH one in testosterone (*p* < 0.0001), Fig. 9 and Table 3.

**Table 3:** Reproductive parameters. Serum concentrations of (a) FSH, (b) LH, and (c) Testosterone in different study groups are presented as mean ± SD.

Variables	Groups				F Test	*P* value
	SH	IR	PC	PRP/PC		
FSH	0.3467 ± 0.03^**a**^	0.1618 ± 0.03^**d**^	0.242 ± 0.02^**c**^	0.302 ± 0.06^**b**^	23.05	< 0.0001
LH	0.3562 ± 0.03^**a**^	0.1372 ± 0.007^**d**^	0.249 ± 0.03^**c**^	0.2642 ± 0.05^**b**^	35.97	< 0.0001
Testosterone	3.827 ± 0.7^**a**^	2.828 ± 0.07^**b**^	2.823 ± 0.09^**b**^	2.958 ± 0.49^**b**^	5.794	< 0.0001

Statistical analysis was performed using ordinary one-way ANOVA followed by a post hoc test with GraphPad software. Different superscript letters (a, b, c, d) indicate statistically significant differences between groups (*p* < 0.05).

Mentioned superscript letters on different groups (raw) are significant at *P* < 0.05 using one-way ANOVA test and Tukey Post hoc test at DFN and DFD equal 3 and 20.

### Hematological analysis

**Fig. 10:**
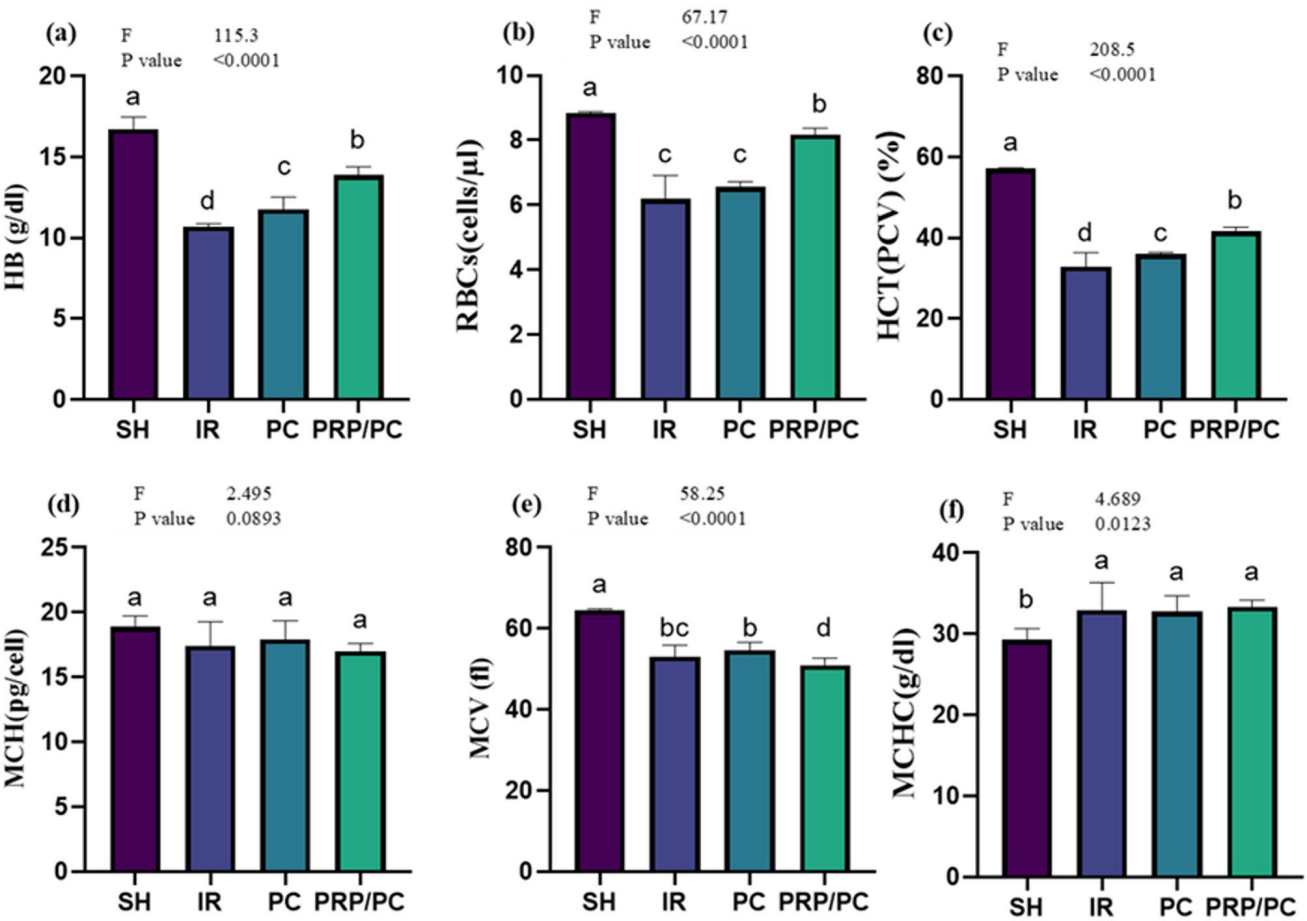
Hematological analysis. Levels of (**a**) HB, (**b**) RBCs, (**c**) HCT (PCV), (**d**) MCV, (**e**) MCH, and (**f**) MCHC in testis homogenates from different study groups are presented as mean ± SD. Statistical analysis was performed using ordinary one-way ANOVA followed by Tukey’s post hoc test with GraphPad software. Different superscript letters indicate statistically significant differences between groups, while the same letters denote no significant difference (*p* > 0.05).

Comparison of hematological parameters between groups is listed in Table 4. Hemoglobin (HB), HCT (PCV), MCV, MCH, and MCHC levels between groups, Fig. 10.

**Table 4:** Levels of HB, RBCs, HCT (PCV), MCV, MCH, and MCHC in different study groups are presented as mean ± SD.

Variables	Groups				F Test	*P* value
	SH	IR	PC	PRP/PC		
HB	16.7 ± 0.76^a^	10.7 ± 0.17^d^	11.75 ± 0.17^c^	13.88 ± 0.5^b^	115.3	< 0.0001
RBCs	8.84 ± 0.03^a^	6.198 ± 0.7^**c**^	6.563 ± 0.15^c^	8.172 ± 0.19^b^	67.17	< 0.0001
HCT(PCV)	57.09 ± 0.22^a^	32.8 ± 3.4^d^	35.87 ± 0.47^c^	41.62 ± 1.04^b^	208.5	< 0.0001
MCV	64.58 ± 0.2^a^	53.02 ± 2.86^bc^	54.69 ± 1.9^b^	50.95 ± 1.7^d^	58.25	< 0.0001
MCH	18.89 ± 0.8^a^	17.43 ± 1.8^a^	17.92 ± 1.4^a^	16.99 ± 0.6^a^	2.495	= 0.0893
MCHC	29.26 ± 1.3^a^	32.92 ± 3.4^a^	32.75 ± 1.96^a^	33.36 ± 0.8^a^	4.689	< 0.0001

Mentioned superscript letters on different groups (raw) are significant at *P* < 0.05 using one-way ANOVA test and Tukey Post hoc test at DFN and DFD equal 3 and 20.

Statistical analysis was performed using ordinary one-way ANOVA followed by Tukey’s post hoc test with GraphPad software. Different superscript letters indicate statistically significant differences between groups, while identical letters denote no significant difference (*p* > 0.05).

## Discussion

Testicular ischemia induces germ cell apoptosis because of hypoxia and intracellular ATP depletion, and metabolic imbalance promotes the accumulation of harmful metabolites. Subsequent reperfusion triggers an abrupt overproduction of ROS, including hydrogen peroxide, superoxide anion, hydroxyl radical, singlet oxygen, hypochlorous acid, and nitric oxide^33^, amplifying oxidative stress and aggravating tissue injury. A high concentration of ROS induces protein coagulation, DNA impairment, and lipid peroxidation in the cellular membrane, causing a reduction in cellular viability^34^. Rich unsaturated fatty acids of the testis make it sensitive to ROS-related cellular injury^35^.

Previous studies have demonstrated that unilateral orchidectomy induces compensatory hypertrophy and functional adaptation in the remaining testis through alterations in gonadotropin feedback mechanisms. However, these adaptive responses develop gradually and become evident only over longer periods^36^, whereas our experimental endpoint of 24 h represents an early stage in which such compensatory effects are likely negligible.

Platelet-rich plasma has been widely investigated as a regenerative therapy in I/R injury across different organ systems. The therapeutic potential of PRP is primarily mediated by its high concentration of growth factors, such as TGF-β1, IGF, and VEGF, which regulate angiogenesis, cellular proliferation, anti-apoptotic signaling, and tissue remodeling. VEGF is markedly upregulated following I/R insult and contributes to vascular restoration and cellular survival. Therefore, the concentrated delivery of these bioactive mediators via PRP enhances tissue repair and attenuates oxidative and inflammatory damage in testicular torsion–detorsion models^20^. In the present study, PRP was not exogenously activated before injection. Instead, platelet activation was allowed to occur endogenously upon contact with tissue collagen and the surrounding microenvironment. This approach is considered to better mimic physiological conditions and promote a more gradual and sustained release of growth factors^37^. This finding is in agreement with *Anitua *et al., who reported that endogenous activation supports a sustained release of growth factors, in contrast to exogenous activation, which induces a rapid initial burst followed by early depletion^38^.

Platelet-rich plasma heterogeneity potentially influences NF-κB and Bax expression even when oxidative stress parameters are improved^39^. Moreover, platelet-derived cytokines are capable of directly activating NF-κB signaling in endothelial and parenchymal cells. Technical factors also influence interpretation: NF-κB activity is best judged by its nuclear translocation or phosphorylation, and IHC for total NF-κB may overrepresent localized inflammatory foci^40^, and platelets can promote thrombo-inflammatory interactions with neutrophils, monocytes, and T cells during ischemia–reperfusion injury. Consequently, NF-κB immunohistochemical staining may partially reflect infiltrating immune cells rather than exclusively resident germ cells^41^.

The choice of a 10 µL intratesticular volume was based on previously published work in rat testicular ischemia–reperfusion models that used the same order of magnitude for local injections and reported both technical feasibility and biological efficacy without mechanical damage to the testis. In particular, prior torsion/detorsion studies have successfully administered antioxidant or cytoprotective agents in very small volumes or doses at the time of detorsion, demonstrating significant protection of testicular structure and function and establishing these parameters as acceptable in this model^22^.

Post conditioning demonstrates a viable surgical option, especially in the early stages of reperfusion when testis damage is frequent^42^. Our study indicated that ten cycles of 10-s reperfusion and 10-s ischemia effectively attenuated testis damage after 24 h. Histological investigation with H&E staining indicated that the sham group had normal testicular architecture, including intact seminiferous tubules and full spermatogenesis, which was consistent with previously described baseline testicular histomorphology in healthy adult rats^43^. In contrast, the I/R group showed severe testicular damage, including vacuolated seminiferous epithelium and degenerated tubules, which is consistent with previous research indicating that oxidative stress and inflammation caused by reperfusion considerably damage germinal epithelium^44^. The increased epithelial thickness seen in the I/R group is attributed to interstitial edema and inflammatory infiltration, which are prevalent during testicular torsion-detorsion^45^.

Interestingly, the PC group exhibited a nearly normal histological architecture comparable to the sham group, suggesting that post-conditioning exerted a partial protective and restorative effect on testicular tissue. In contrast, the PRP/PC group demonstrated heterogeneous histological findings, where some seminiferous tubules showed nearly complete spermatogenesis, while others contained necrotic cells within constricted lumina, indicating variable and uneven regenerative responses. This variability may reflect differences in local microenvironmental conditions and responsiveness to treatment^46^.

The observed reduction in seminiferous tubule diameter in the PRP/PC group can be interpreted in the context of ongoing tissue remodeling, where residual degenerative changes and early fibrotic processes may contribute to tubular compaction. It is important to note that tubular diameter alone is not a definitive indicator of spermatogenic function, particularly in post-injury states, and should be interpreted alongside cellular and structural parameters. This is consistent with the original description of the Johnsen scoring system, which emphasizes germ cell maturation rather than morphometric parameters^31^. Collectively, these findings suggest that while PRP has regenerative potential, its effects are not uniformly predictable, and post-conditioning alone may provide more consistent structural preservation^47^.

Interestingly, the PC group had a nearly normal histological appearance, similar to the sham group, indicating that the post-conditioning treatment had a partial protective effect. The histology results in the PRP/PC group were varied. While some tubules had virtually full spermatogenesis, others indicated necrotic cells inside restricted lumens, indicating uneven regeneration responses. The total decline in tubule diameter in this group was attributed to remaining degenerative alterations and fibrosis. This agrees with Previous studies, which have indicated that PRP includes many growth factors, but its therapeutic effectiveness is extremely dose-dependent and may vary depending on tissue state and delivery timing^48,49^.

The paradoxical finding of reduced oxidative stress alongside increased NF-κB and Bax in the PRP/PC group can be explained by the time-dependent and biphasic nature of PRP signaling. PRP contains a complex secretome of growth factors, cytokines, chemokines, and platelet receptors that can transiently activate inflammatory and survival pathways early, before promoting antioxidant and reparative responses later. This early phase is capable of inducing a short-term rise in NF-κB and Bax even when downstream oxidative markers subsequently improve. Platelet-derived mediators can recruit and activate endothelial and immune cells, producing an initial inflammatory spike that elevates NF-κB and its downstream apoptotic signals^39^.

Transient NF-κB activation has also been documented downstream of PRP releasate through PI3K–AKT signaling, reflecting an early pro-inflammatory or pro-survival transcriptional program^40^. Consistently, several studies report that PRP or platelet lysate treatments result in later reductions in MDA/TBARS and increases in SOD/GSH despite early inflammatory activation^50^^,^^51^. The overall pattern of Bax and NF-κB expression is strongly influenced by timing, PRP formulation, platelet activation state, and dosing. Small changes in the time of administration relative to ischemia can shift PRP’s effect from anti-apoptotic to apparently pro-inflammatory, as shown in models where PRP given before ischemia produced stronger protective outcomes than when applied at reperfusion^52^.

Likewise, Bax upregulation can be transient or reflect selective apoptosis of irreversibly damaged cells during tissue remodeling, rather than widespread apoptotic injury^53^. Methodological heterogeneity across studies using IHC, Western blot, or qPCR can also yield variable Bax/NF-κB profiles, with in vivo IHC particularly sensitive to immune cell recruitment.^54^^,^^55^. Therefore, a single-timepoint elevation of Bax or NF-κB after PRP administration should be interpreted with caution, as it does not necessarily indicate net tissue loss; instead, it may reflect an early adaptive inflammatory phase within a broader reparative trajectory^50^.

The present study revealed that red blood cell parameters, including Hb, g/dL, HCT, and MCV, were significantly decreased in the (I/R) group, while they were partially restored in the treatment groups compared to the SH group. No significant differences were observed in (MCH) and (MCHC). These findings are in agreement with Yildiz et al.^56^, who reported that low-dose sildenafil citrate significantly reduced RBC count and MCV (*p* < 0.05). Conversely, previous studies indicated no significant changes in the hematological profile of individuals with testicular torsion^57^.

Regarding reproductive hormones, testicular I/R injury significantly decreased serum FSH and LH levels, whereas testosterone levels remained unchanged compared to the SH group. These hormonal alterations were partially improved in rats treated with PC alone, as well as in those receiving the combined PRP/PC treatment, although values remained lower than in the SH group. Consistently, recent research demonstrated that administration of L-citrulline modulates reproductive hormone levels and alleviates I/R-induced testicular injury^58^.

The present study showed MDA’s elevation in the I/R group, indicating the cellular damage resulted in testicular ischemia reperfusion, despite a reduction in the treatment groups, PC and PRP/PC, in comparison with the SH group. Furthermore, an increase in antioxidant enzymes CAT and GSH in PC and PRP/PC proved the protective effect of platelet-rich plasma with post-conditioning against I/R-I on the testis, which was in agreement with the protection against testicular I/R via administration of Alpha-Glucosyl Hesperidin and Procyanidin alone or in combination^59^. On the other hand, the latter proves that proinflammatory cytokines such as TNF alpha and IL6 were increased at I/R due to tissue damage occurring^20^. which agrees with the recent work. PC, especially with PRP, ameliorates the testicular inflammation resulting from I/R injuries. Additionally, programmed cellular apoptosis indicated via Caspase 3 was markedly reduced after treatment than before, as well as compared with the sham group. HSP70 is a homeostatic enzyme released in response to a testicular injury. It stimulates spermatogenesis and preserves cells from apoptosis^36^.

The current study found that HSP70 was decreased in I/R in comparison to SH and in treatment with PC alone or with PRP, which further supported the findings of^46^. eNOS controls vascular pressure by producing nitric oxide, which is crucial for endothelial function^60^.I/R reduced testicular eNOS in comparison with SH, despite its elevation in the case of treatment groups, proving that PRP and PC protect the testicular circulation from IR-I. VEGF encourages angiogenesis and stimulates steroidogenic cells, including testicular Leydig cells. Likewise, VEGF maintains the testicular blood vessels’ permeability^30^, which was matched with this work showing an elevation of VEGF in testicular tissues after treatment, other than its reduction in IR only, compared with sham rats.

## Conclusion

The results of the present study indicated that although PRP with PC did not fully reverse the damage, it protects against I/R harm in the testicular torsion model, decreases oxidative stress, apoptosis, and inflammation, and maintains cell shape.

### Study limitations

Although Johnsen’s index and histological scoring provide insight into seminiferous tubular integrity, they do not confirm functional spermatogenic recovery. The absence of functional assessments (e.g., epididymal sperm count, motility, viability, and chromatin integrity) limits interpretation of the findings; therefore, the observed improvements should be considered indicative of partial structural restoration rather than definitive recovery of fertility.

Furthermore, although PRP has shown protective effects against testicular ischemia–reperfusion injury in animal models, its ability to achieve complete biochemical normalization and sustained functional recovery remains uncertain, particularly in humans. The lack of a PRP-only group also limits differentiation between independent and combined treatment effects. Finally, unilateral orchidectomy, although adopted to reduce animal use and allow intra-subject comparison, may influence hormonal balance and induce compensatory changes in the contralateral testis. These factors should be considered when interpreting the results.

## Supplementary Information

Below is the link to the electronic supplementary material.

Supplementary Material 1

## Data Availability

No datasets were generated or analyzed during the current study.

## Abbreviations

TT: Testicular torsion
I/R: Ischemia–reperfusion
IR-I: Ischemia–reperfusion injury
PRP: Platelet-rich plasma
PC: Post-conditioning
LT: Left testis
i.t: Intratesticular
i.p: Intraperitoneally
MDA: Malondialdehyde
CAT: Catalase
GSH: Reduced glutathione
eNOS: Endothelial nitric oxide synthase
HSP70: Heat shock protein 70
VEGF: Vascular endothelial growth factor
TNF-α: Tumor necrosis factor alpha
IL-6: Interleukin-6
NF-κB: Nuclear factor kappa B
Bax: Bcl-2-associated X protein
ROS: Reactive oxygen species
PAS: Periodic acid–Schiff
H&E: Hematoxylin and eosin
Hb: Hemoglobin
RBCs: Red blood cells
HCT (PCV): Hematocrit (packed cell volume)
MCV: Mean corpuscular volume
MCH: Mean corpuscular hemoglobin
MCHC: Mean corpuscular hemoglobin concentration
