# Supplementary figures and images for: Platelet-rich plasma enhances post-conditioning recovery from testicular ischemia–reperfusion injury: a novel experimental approach
Source: Sci Rep. 2026 Apr 17;16:12709. doi: 10.1038/s41598-026-46712-6 (PMC13090401; doi:10.1038/s41598-026-46712-6)

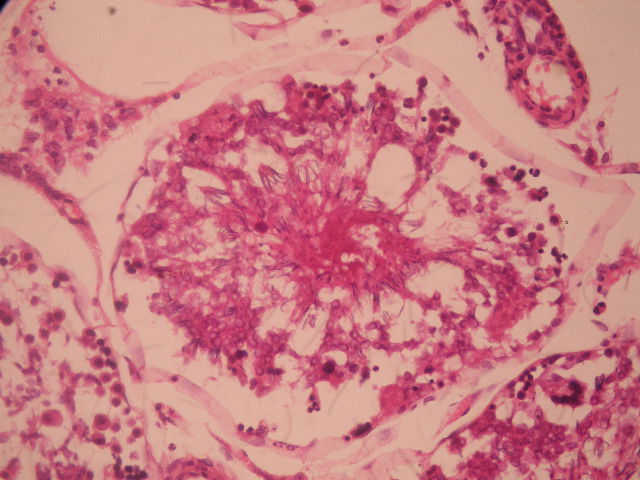

Supplement: Supplementary file 1 — Supplementary Material 1 [file 41598_2026_46712_MOESM1_ESM.zip › Raw image/HE/IR x40.JPG]

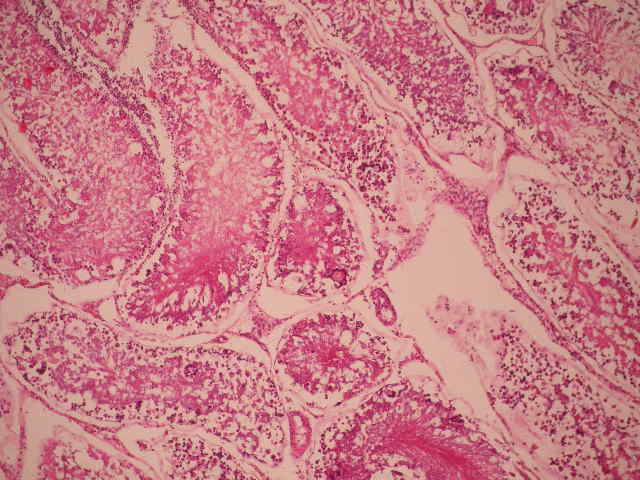

Supplement: Supplementary file 1 — Supplementary Material 1 [file 41598_2026_46712_MOESM1_ESM.zip › Raw image/HE/IR.JPG]

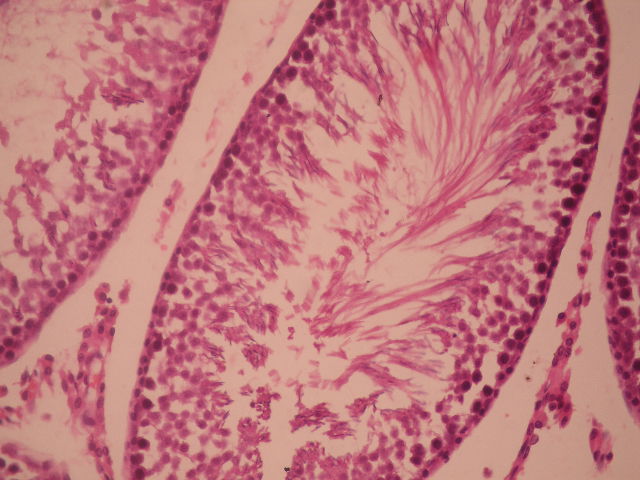

Supplement: Supplementary file 1 — Supplementary Material 1 [file 41598_2026_46712_MOESM1_ESM.zip › Raw image/HE/PC x40.JPG]

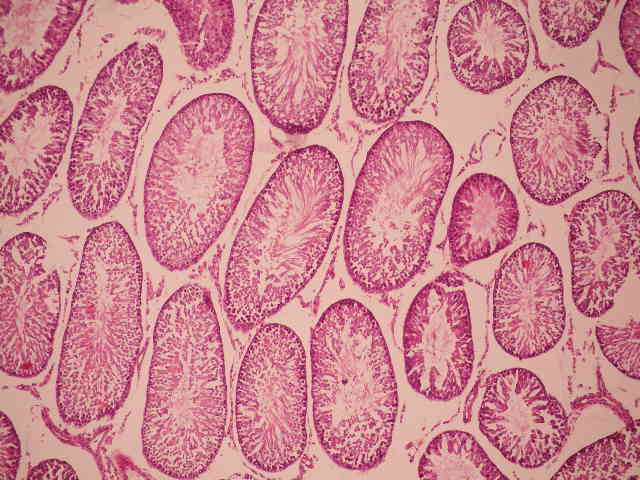

Supplement: Supplementary file 1 — Supplementary Material 1 [file 41598_2026_46712_MOESM1_ESM.zip › Raw image/HE/PC.JPG]

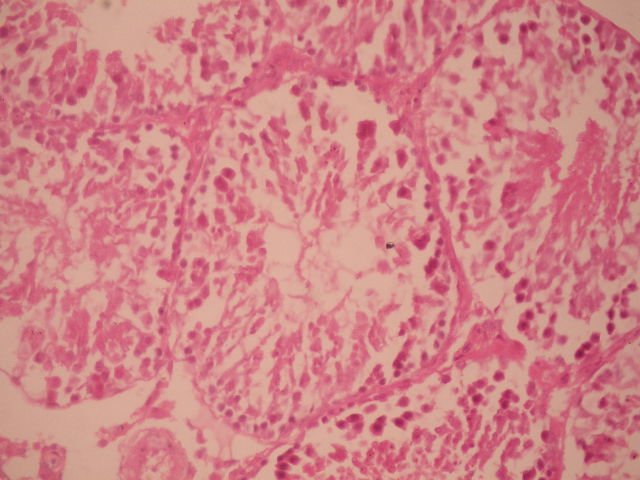

Supplement: Supplementary file 1 — Supplementary Material 1 [file 41598_2026_46712_MOESM1_ESM.zip › Raw image/HE/PRP+PC x40.JPG]

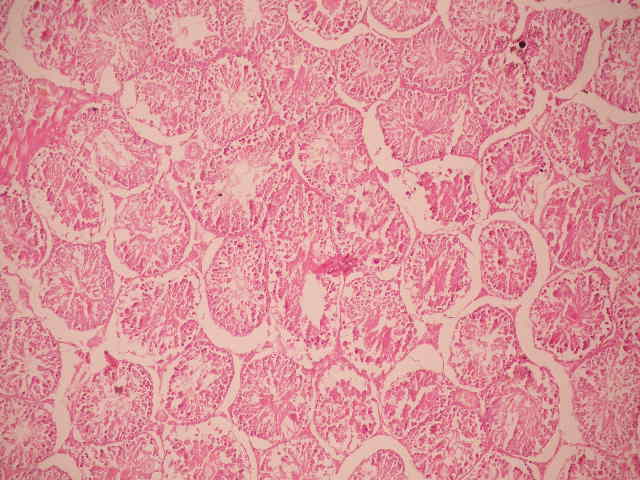

Supplement: Supplementary file 1 — Supplementary Material 1 [file 41598_2026_46712_MOESM1_ESM.zip › Raw image/HE/PRP+PC.JPG]

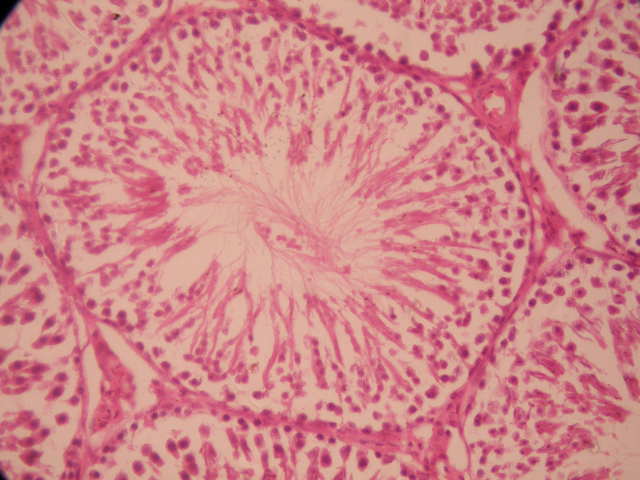

Supplement: Supplementary file 1 — Supplementary Material 1 [file 41598_2026_46712_MOESM1_ESM.zip › Raw image/HE/Sham x40.JPG]

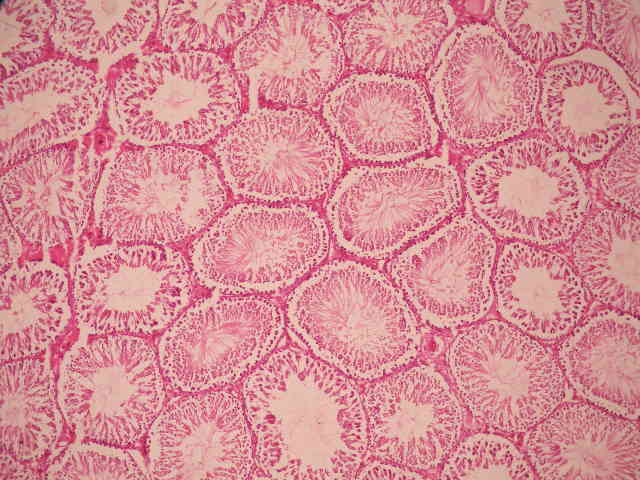

Supplement: Supplementary file 1 — Supplementary Material 1 [file 41598_2026_46712_MOESM1_ESM.zip › Raw image/HE/Sham.JPG]

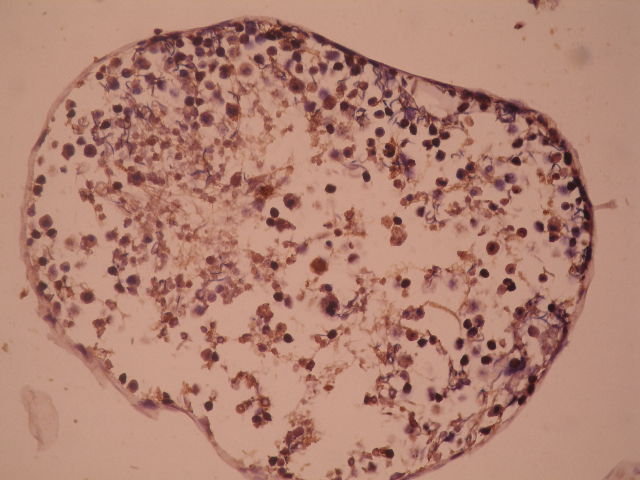

Supplement: Supplementary file 1 — Supplementary Material 1 [file 41598_2026_46712_MOESM1_ESM.zip › Raw image/Immuno bax/IR.JPG]

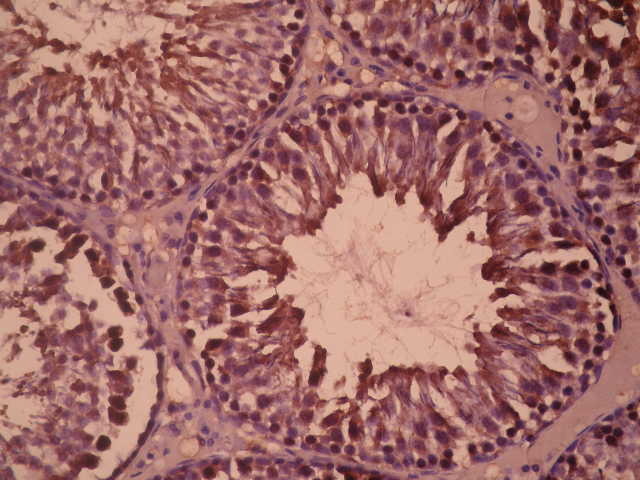

Supplement: Supplementary file 1 — Supplementary Material 1 [file 41598_2026_46712_MOESM1_ESM.zip › Raw image/Immuno bax/PC.JPG]

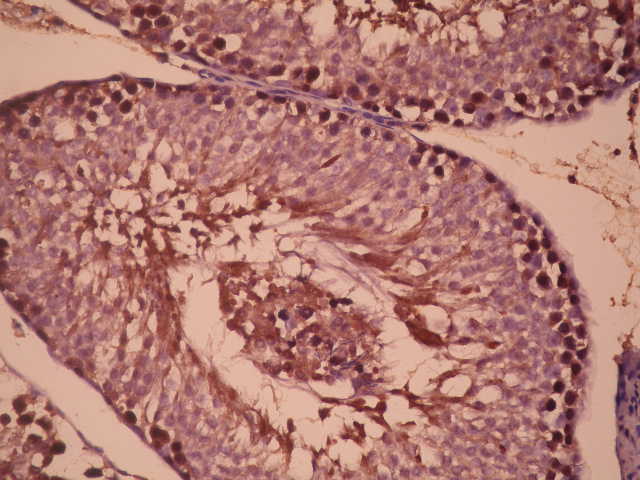

Supplement: Supplementary file 1 — Supplementary Material 1 [file 41598_2026_46712_MOESM1_ESM.zip › Raw image/Immuno bax/PRP+PC.JPG]

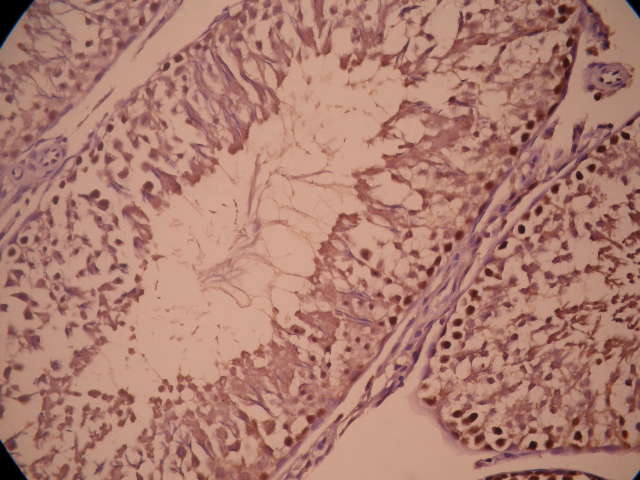

Supplement: Supplementary file 1 — Supplementary Material 1 [file 41598_2026_46712_MOESM1_ESM.zip › Raw image/Immuno bax/Sham.JPG]

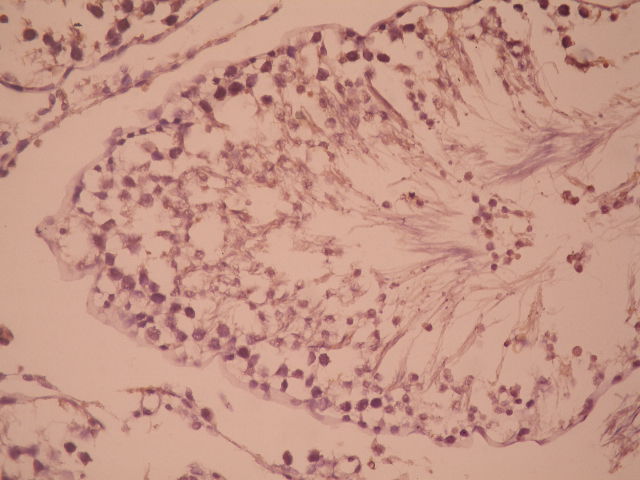

Supplement: Supplementary file 1 — Supplementary Material 1 [file 41598_2026_46712_MOESM1_ESM.zip › Raw image/immuno nf-kb/IR.JPG]

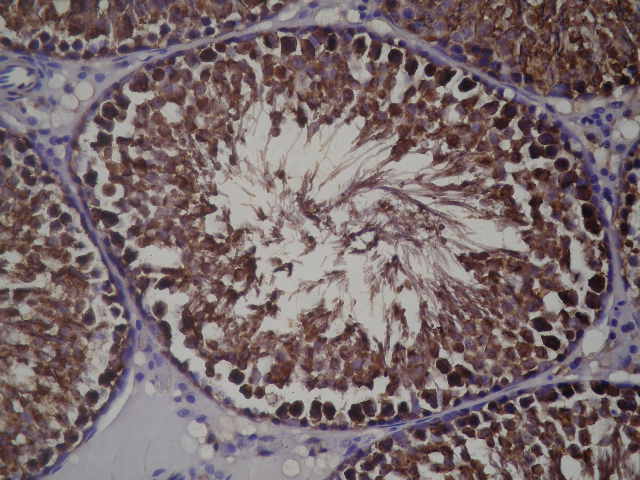

Supplement: Supplementary file 1 — Supplementary Material 1 [file 41598_2026_46712_MOESM1_ESM.zip › Raw image/immuno nf-kb/PC.JPG]

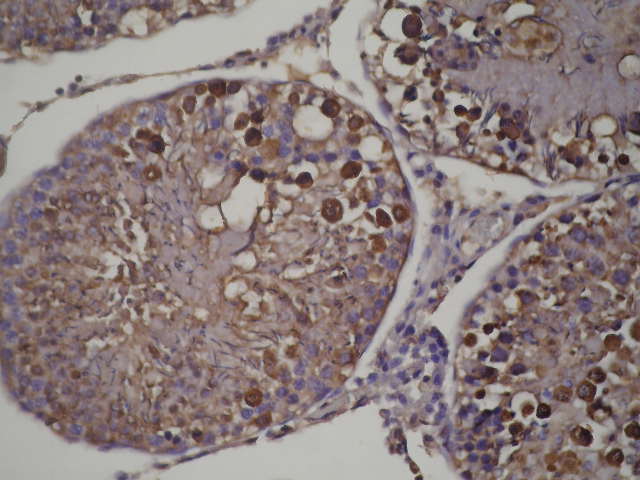

Supplement: Supplementary file 1 — Supplementary Material 1 [file 41598_2026_46712_MOESM1_ESM.zip › Raw image/immuno nf-kb/PRP+PC.JPG]

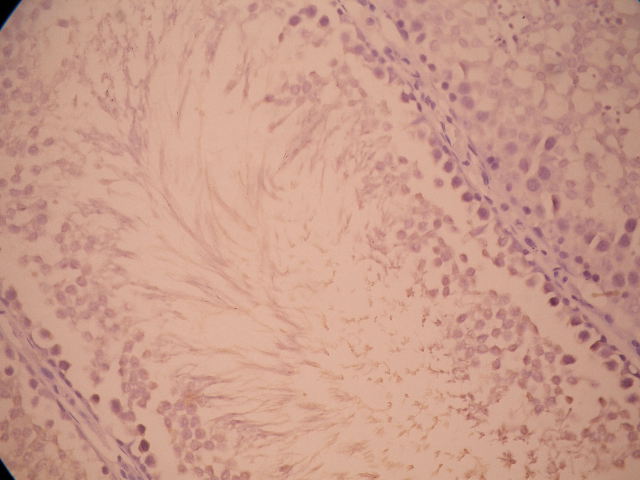

Supplement: Supplementary file 1 — Supplementary Material 1 [file 41598_2026_46712_MOESM1_ESM.zip › Raw image/immuno nf-kb/Sham.JPG]

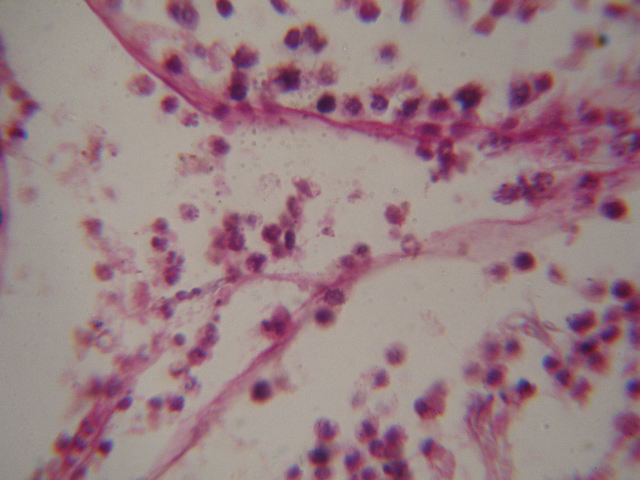

Supplement: Supplementary file 1 — Supplementary Material 1 [file 41598_2026_46712_MOESM1_ESM.zip › Raw image/PAS/IR x100.JPG]

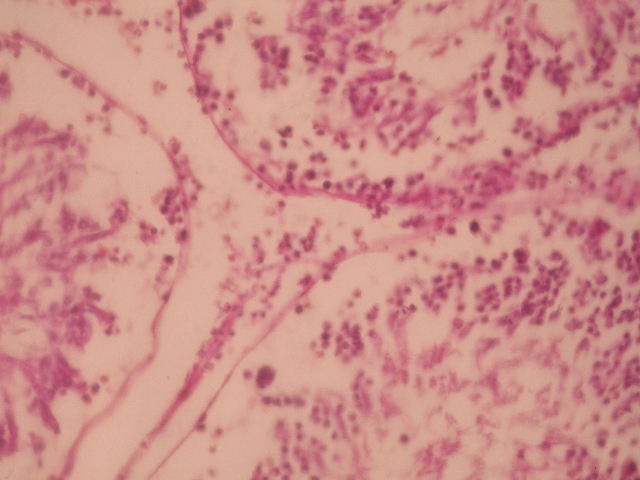

Supplement: Supplementary file 1 — Supplementary Material 1 [file 41598_2026_46712_MOESM1_ESM.zip › Raw image/PAS/IR.JPG]

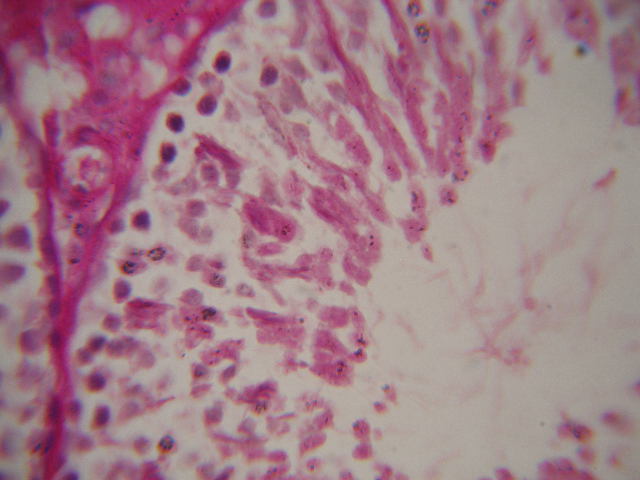

Supplement: Supplementary file 1 — Supplementary Material 1 [file 41598_2026_46712_MOESM1_ESM.zip › Raw image/PAS/PC x100.JPG]

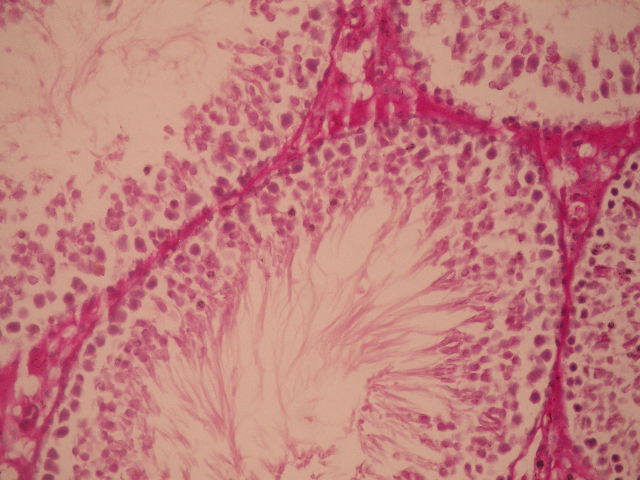

Supplement: Supplementary file 1 — Supplementary Material 1 [file 41598_2026_46712_MOESM1_ESM.zip › Raw image/PAS/PC.JPG]

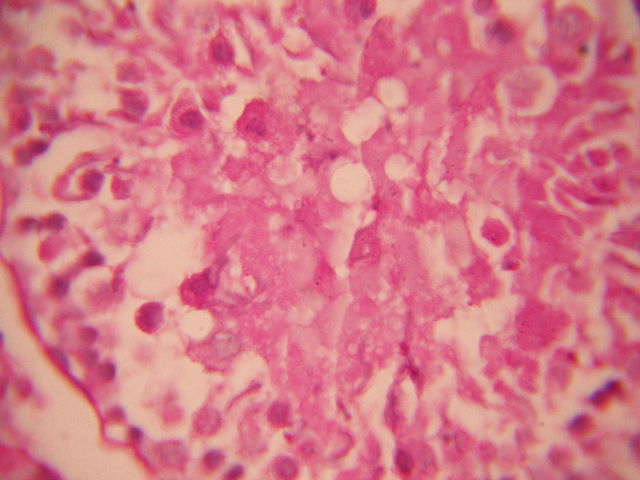

Supplement: Supplementary file 1 — Supplementary Material 1 [file 41598_2026_46712_MOESM1_ESM.zip › Raw image/PAS/PRP+PC x100.JPG]

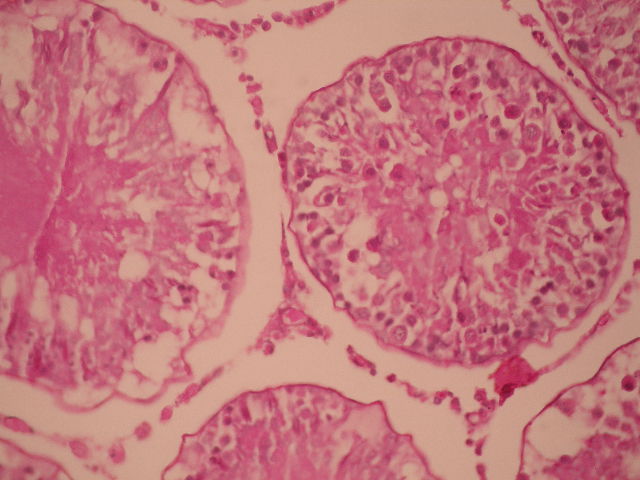

Supplement: Supplementary file 1 — Supplementary Material 1 [file 41598_2026_46712_MOESM1_ESM.zip › Raw image/PAS/PRP+PC.JPG]

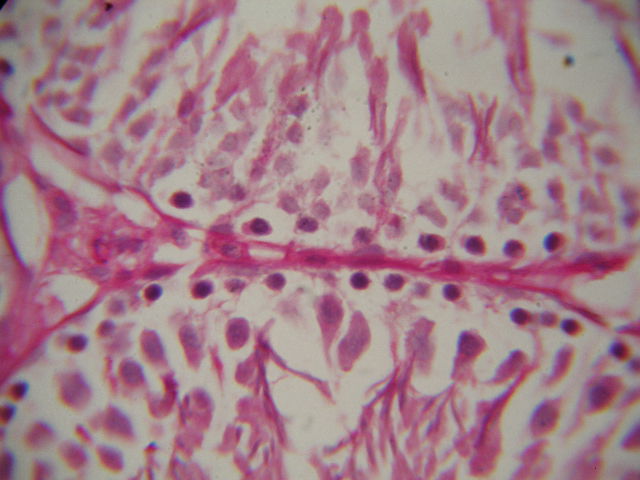

Supplement: Supplementary file 1 — Supplementary Material 1 [file 41598_2026_46712_MOESM1_ESM.zip › Raw image/PAS/Sham x100.JPG]

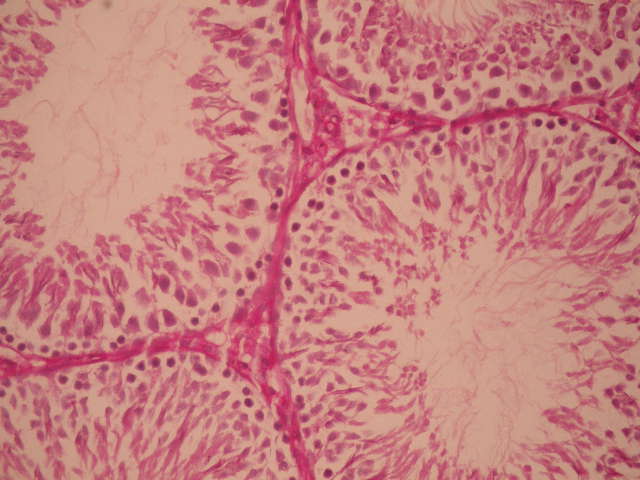

Supplement: Supplementary file 1 — Supplementary Material 1 [file 41598_2026_46712_MOESM1_ESM.zip › Raw image/PAS/Sham.JPG]
